# Pathotype and Genetic Diversity amongst Indian Isolates of *Xanthomonas oryzae* pv. *oryzae*

**DOI:** 10.1371/journal.pone.0081996

**Published:** 2013-11-29

**Authors:** Deo Mishra, Manne Ramachander Vishnupriya, Madhusoodana Girija Anil, Kotilingam Konda, Yog Raj, Ramesh V. Sonti

**Affiliations:** 1 Bayer, BioScience, Hyderabad, India; 2 CSIR-Centre for Cellular and Molecular Biology, Hyderabad, India; St. Petersburg Pasteur Institute, Russian Federation

## Abstract

A number of rice resistance genes, called *Xa* genes, have been identified that confer resistance against various strains of *Xanthomonas oryzae* pv. *oryzae* (Xoo), the causal agent of bacterial blight. An understanding of pathotype diversity within the target pathogen population is required for identifying the *Xa* genes that are to be deployed for development of resistant rice cultivars. Among 1024 isolates of Xoo collected from 20 different states of India, 11 major pathotypes were distinguished based on their reaction towards ten *Xa* genes (*Xa1*, *Xa3*, *Xa4*, *xa5*, *Xa7*, *xa8*, *Xa10*, *Xa11*, *xa13*, *Xa21*). Isolates belonging to pathotype III showing incompatible interaction towards *xa8*, *xa13* and *Xa21* and compatible interaction towards the rest of *Xa* genes formed the most frequent (41%) and widely distributed pathotype. The vast majority of the assayed Xoo isolates were incompatible with one or more *Xa* genes. Exceptionally, the isolates of pathotype XI were virulent on all *Xa* genes, but have restricted distribution. Considering the individual R-genes, *Xa21* appeared as the most broadly effective, conferring resistance against 88 % of the isolates, followed in decreasing order by *xa13* (84 %), *xa8* (64 %), *xa5* (30 %), *Xa7* (17 %) and *Xa4* (14 %). Fifty isolates representing all the eleven pathotypes were analyzed by southern hybridization to determine their genetic relatedness using the IS*1112* repeat element of Xoo. Isolates belonging to pathotype XI were the most divergent. The results suggest that one RFLP haplotype that is widely distributed all over India and is represented in strains from five different pathotypes might be an ancestral haplotype. A rice line with *xa5, xa13* and *Xa21* resistance genes is resistant to all strains, including those belonging to pathotype XI. This three gene combination appears to be the most suitable *Xa* gene combination to be deployed in Indian rice cultivars.

## Introduction

*Xanthomonas oryzae* pv. *oryzae* (Xoo) is the causal agent of bacterial blight, a serious disease of rice. Bacterial blight is endemic and causes serious yield losses for the rice crop grown in irrigated, low land areas across Asia. Host plant resistance is the most effective way of managing yield losses due to the disease as chemical control is not effective [1]. Almost thirty different rice genes (called *Xa* genes) that confer resistance against various races and pathotypes of Xoo have been identified [2]. Many of these resistance genes have been tagged with closely linked molecular markers and are being used in marker assisted selection [3–6]. These resistance genes display specificity with regard to their effectiveness against different pathogen races. Therefore knowledge of the pathotype diversity in the target pathogen population is essential for making an informed choice of resistance genes that are to be used in a breeding program.

In India, bacterial blight occurs in a large number of states with yield losses going up to 60-80% in severe infections. Pathotype studies conducted in India under the All India Coordinated Rice Improvement Project (AICRIP) during the 1970s and 1980s indicated that pathotypes Ia and Ib were distributed all over the country [7,8]. These two pathotypes exhibit similar reactions on differential cultivars and are characterized by incompatibility with rice varieties BJ1 (*xa5* and *xa13*) and DV85 (*xa5*, *Xa7* and *xa24*). They are distinguished from each other on the basis of reaction pattern on rice cultivar IR20 which carries the *Xa4* resistance gene; pathotype Ia strains are incompatible on IR20 while pathotype Ib strains are compatible. IS*1112*, a repeat element native to Xoo, is a good probe for genotyping different strains of the pathogen as it is present in multiple copies and reveals substantial inter-strain variability [9–12]. Yashitola et al. (1997) performed pathotype analysis and DNA fingerprinting studies with IS*1112* on a set of Indian isolates of Xoo that were collected between 1991 to 1995 [13]. They observed that the vast majority of strains (60/67) analyzed were incompatible with BJ1 and DV85 but were compatible with IR20. This suggested that all of these strains belonged to pathotype Ib and none were like pathotype Ia. RFLP haplotyping demonstrated that strains belonging to this pathotype clustered together in a dendrogram as a lineage comprised of closely related strains. The BXO1 Xoo strain was considered as the type strain for this lineage. The remaining strains (7/67) were found to be compatible with BJ1 and DV85 (therefore they were neither pathotype Ia nor pathotype Ib) and they had very diverse haplotypes. The BXO8 strain was considered as an example of this group of strains. Shanti et al. (2001) inoculated a collection of Xoo strains from Eastern India on a set of near-isogenic rice lines containing any one of several *Xa* genes in the IR24 rice varietal background [14]. They identified several Xoo strains that are compatible with the *xa13* disease resistance gene and suggested that the combination of *Xa4*, *xa5* and *Xa21*, when pyramided, would be effective against strains from Eastern India. Lore et al. (2011) inoculated 224 Xoo strains collected between 1999-2006 from the North western Indian state of Punjab on near-isogenic rice lines containing individual *Xa* genes and found that none of these strains were compatible with the *xa13* disease resistance gene [15]. Approximately 93% of the isolates were incompatible with *Xa21* while 95% of the isolates were found to be compatible with the *xa5* resistance gene. In an earlier study of Xoo strains from the Punjab, several isolates were found to be compatible with *xa13* [16]. 

All of these above mentioned studies were done with either a limited number of strains or were from one particular geographic area of India. In order to obtain a more comprehensive picture of the pathotype diversity of Xoo in India, we have collected 1024 isolates from twenty different states. These strains were inoculated on a set of near-isogenic lines containing any one of nine *Xa* genes which have been used in earlier studies to assess pathotype diversity of Indian strains of Xoo (14,15). Besides these lines, the IR8 rice variety which carries the *Xa11* disease resistance gene was also included in the study. Thus, the effectiveness of ten different *Xa* genes was assessed and eleven different pathotypes were identified in this study. Representative strains from each of the pathotypes were subjected to DNA fingerprinting using the IS*1112* probe and the relationship between RFLP haplotype and pathotype was analysed. The three gene combination of *Xa21, xa13* and *xa5* appears to be appropriate for development of bacterial blight resistant rice varieties in India.

## Materials and Methods

### Bacterial collection and maintenance

**Figure 1:**
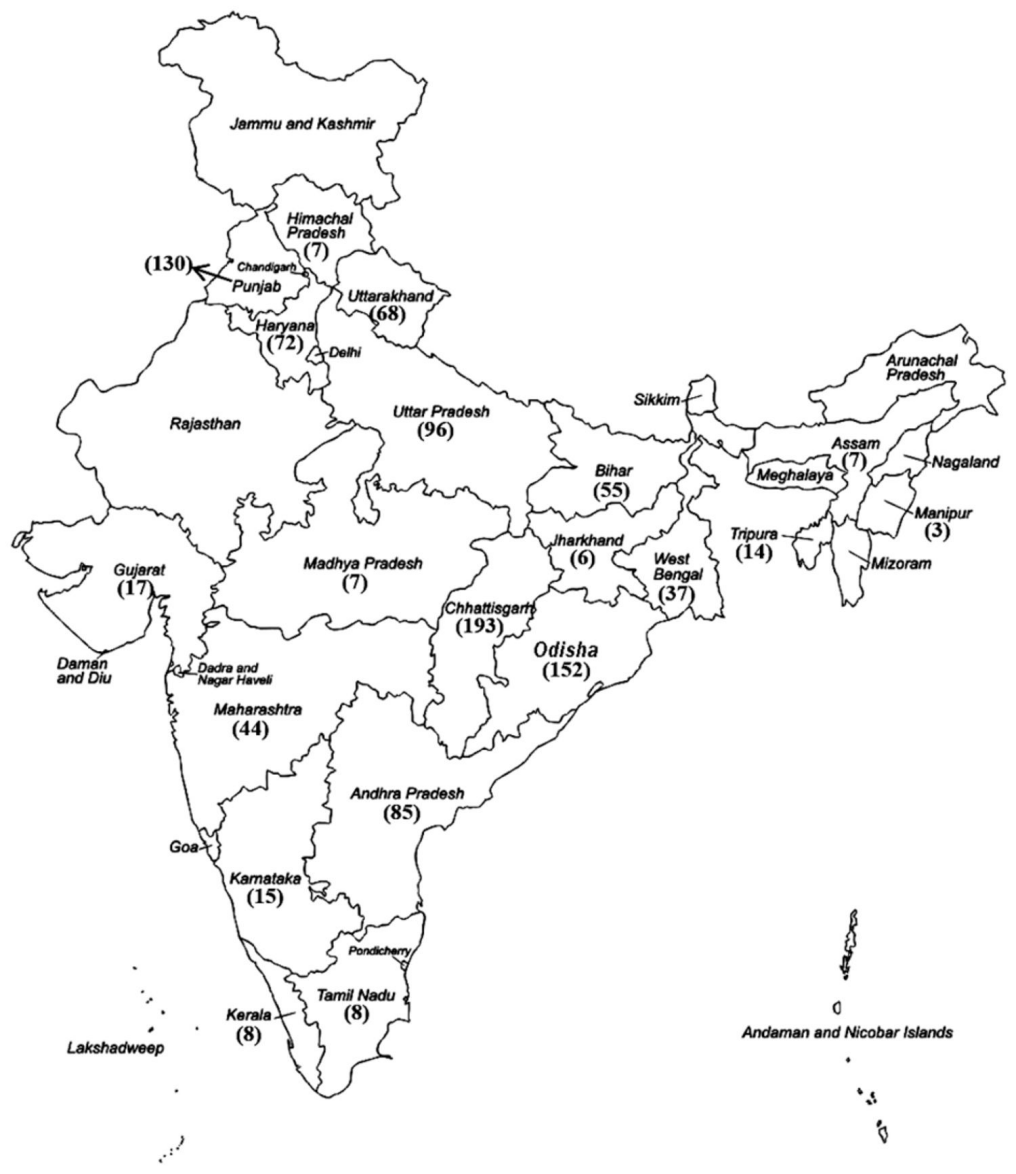
Statewise distribution of *X. oryzae* pv. *oryzae* strains collected from India. *X. oryzae* pv. *oryzae* strains were collected from different states in India. The numbers in parentheses indicate the number of strains isolated from each state. Overall, the states from which more isolates were collected are those that have been more extensively surveyed.

 Infected leaf samples were collected during 2004 - 2012, from different rice growing states of India representing various geographical locations (Figure 1). Although a majority of the states were sampled, because of constraints arising from the fact that a vast region was involved, certain states were sampled sparsely or were not sampled at all. All the samples were collected from private rice fields with owner’s permission. Samples were placed in auto seal plastic packets with silica gel and stored in a refrigerator until isolation of the pathogen. Single colonies of cultures isolated from disease samples were picked up from Peptone Sucrose Agar (PSA) plates, maintained in liquid medium at 4°C for routine work and in 20% glycerol at -80°C for long term storage. 

### Pathotype analysis

 The seeds of bacterial blight differential rice lines (IRBB-1, 3, 4, 5, 7, 8, 10, 13, 21, IR8), the susceptible check line (IR24) and 7 different gene pyramid lines (IRBB-52, 54, 55, 57, 58, 59, and 60) were provided by the International Rice Research Institute (IRRI), Philippines. The plants were grown in plastic trays (55 x 40 x 15 cm) in a greenhouse. The trays were filled with a mixture of soil and farmyard manure at a ratio of 3:1. N-P-K were supplied to the plants at the rate of 100-75-74 kg/ha as the basal dose in the form of urea, superphosphate, and muriate of potash. Sowing of the differentials was done at 20 days intervals to get 2 stages of plants (60 and 40 days old) at the time of inoculation. The trays were irrigated every day. Adequate plant protection measures were taken to ensure healthy and vigorous growth of the plants. Plants were clip-inoculated with bacterial suspensions of 10^9^ cfu/ml [17]. Four leaves per plant were inoculated for each isolate-cultivar combination for 60 and 40 days old plants.

Disease observations were taken 14 days after inoculation by measuring lesion length. Lesion lengths <5 cm were considered as resistant, 5-10 cm were considered as moderately resistant and >10 cm were considered as susceptible. Pathotype grouping was done based on the reaction pattern onto the differentials.

### Genotype analysis

 A total of 50 Xoo isolates representing all pathotypes were used for genomic DNA isolation and fingerprinting. Two previously genotyped strains from the study of Yashitola et al. (1997), BXO1 and BXO8, were also included in the present analysis for comparison.

### DNA isolation and Southern hybridization

 Xoo cultures were grown for 24 h in peptone sucrose medium on a rotary shaker at 28°C. Genomic DNA was isolated using the phenol-chloroform method. Genomic DNA was digested using restriction endonucleases *Eco*RI (New England Biolabs Inc., Ipswich, MA, USA) for 1 h at 37°C. One microgram of completely digested DNA from each strain was separated by electrophoresis on 0.7% agarose gels, denatured, neutralized, and vacuum-transferred to Hybond N+ (GE Healthcare Bio-Sciences, Uppsala, Sweden) membranes according to the procedure given by Sambrook et al [18]. Blots were pre-hybridized in a solution of 0.5 M sodium phosphate (pH 7.2), 7% sodium dodecyl sulfate (SDS), 1% bovine serum albumin, and 1 mM EDTA for 3 h at 65°C. Probes were labelled with α^32^P-dATP and hybridized for 18 h at 65°C with constant shaking. Blots were washed three times (20 min/wash) at 65°C first with 2× SSC (1× SSC is 0.15 M NaCl plus 0.015 M sodium citrate, pH 7.0), 0.1% SDS, and 5 mM sodium phosphate (pH 7.0) and subsequently with 0.5× SSC, 0.1% SDS, and 3 mM sodium phosphate buffer (pH 7.0). Autoradiography was done with X-ray film. A 1 kilobase DNA ladder (New England Biolabs Inc.) was added to all the gels as a marker. After transfer, the membrane was cut, and the lane carrying the marker was hybridized separately.

### RFLP analysis

 Twenty nine different fragments present between 1 kb and 5 kb were used for genotyping. The haplotype (fragmenting pattern) of each strain obtained by Southern hybridization was compared with all the other strains, and presence or absence of a particular fragment was recorded as 1 and 0, respectively. Similarity matrix was prepared using the dice coefficient option and dendrograms were prepared using the UPGMA (unweighted pair group method of averages) option of the software FREETREE [19]. The confidence limits of dendrograms were determined by bootstrap analysis with 2,000 replications using the same program. The bootstrap values are expressed as a percentage of these 2,000 replications. The dendrogram was drawn using the software TreeView [20]. 

Using the same data, a minimum spanning tree (MST) was also constructed for phylogenetic analysis using the MST gold software programme available at www.bellinghamresearchinstitute.com /software [21]. The pair wise distances between strains were calculated using the equidistant method option in the software programme [21]. A 1000 bootstrap iterations were done on 500 unique MSTs and the MST with highest average bootstrap percentage was taken as the representative MST. The MST was visualized using the software GVEdit for Graphviz version 1.01 [22]. Using the same software, a minimum spanning consensus network was also made, using the pathotyping data in Table 1, for the eleven pathotypes identified in this study. A binary score was generated by taking either a resistant or a moderately resistant interaction as 1 and a susceptible interaction as 0.

**Table 1:** Pathotyping of Indian isolates of *X*. *oryzae* pv. *oryzae*.

Host Differential^b^	*X*. *oryzae* pv. *oryzae* pathotypes ^a^										
	I	II	III	IV	V	VI	VII	VIII	IX	X	XI
**IRBB1 (*Xa1*)**	S	S	S	S	S	S	S	S	S	R, M	S
**IRBB3 (*Xa3*)**	S	S	S	S	S	S	S	S	S	R	S
**IRBB4 (*Xa4*)**	S	S	S	S	R, M	M	R, M	R, M	S	R	S
**IRBB5 (*xa5*)**	S	S	S	S	R, M	R	R	R	R	R, M	S
**IRBB7 (Xa7)**	S	S	S	S	S	S	S	R	R	R, M	S
**IRBB8 (*xa8*)**	S	S	R, M	R, M	R, M	R	R, M	S	S	R	S
**IRBB10 (*Xa10*)**	S	S	S	S	S	S	S	S	S	R, M	S
**IRBB13 (*xa13*)**	R	R	R	R	R	S	S	S	S	R, M	S
**IRBB21 (*Xa21*)**	R, M	S	R, M	S	R	M	R, M	R	R, M	R	S
**IR8 (*Xa11*)**	S	S	S	S	S	R	S	S	S	R, M	S
**IR24**	S	S	S	S	S	S	S	S	S	R, M	S

^a^ Based on mean lesion lengths, the nature of responses were classified into susceptible (S) (above 10 cm), moderately resistant (MR) (between 5-10 cm) and resistant (R) (up to 5 cm). The data presented are from experiments with 60 day old plants; similar results were obtained from inoculation of 40 day old plants. R, M is indicated when some isolates in the pathotype give a resistant reaction while other isolates give a moderately resistant reaction

^b^ The *Xa* resistance gene in each host differential is given in parenthesis. IR8 is a cultivar with the *Xa11* resistance gene and IR24 is the susceptible control.

## Results

### Pathotyping of *X. oryzae* pv. *oryzae* strains

**Figure 2:**
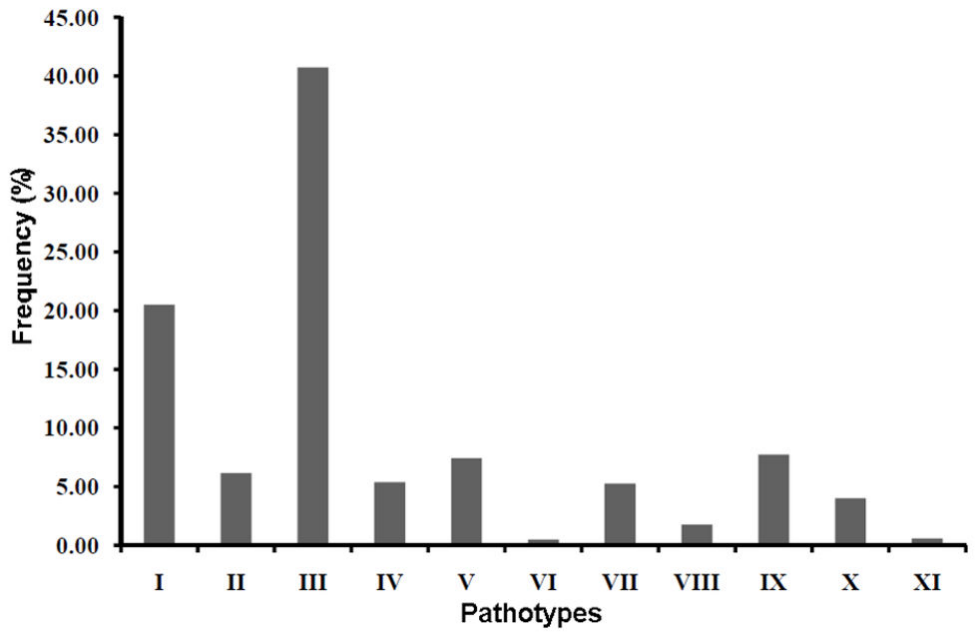
Frequency of *X. oryzae* pv. *oryzae* pathotypes in India. Eleven different pathotypes, designated as I to XI, were identified in this study. The x-axis indicates pathotype and the y-axis indicates % frequency amongst the 1024 isolates that were collected and analyzed.

A total of 1024 isolates of Xoo were collected from 20 different states in India (Figure 1) during 2004 to 2012. All of these isolates were pathotyped using 9 NILs harbouring Xoo resistance genes *Xa1*, *Xa3*, *Xa4*, *xa5*, *Xa7*, *xa8*, *Xa10*, *xa13*, *Xa21* and one variety (IR8) that is reported to carry the *Xa*11 resistance gene. This analysis revealed 11 distinct pathotypes (Table 1; Figure 2). The pathotype III was the most frequent pathotype and accounted for 40.7 % of the isolates. Strains belonging to this pathotype were widely distributed throughout India with presence in 19/20 sampled states in the country (Table 2). Pathotype I was the second most prevalent pathotype comprising 20.5 % of the isolates that were analyzed. This pathotype was present in 14 of the states that were covered in this study. As compared to these two pathotypes, the remaining nine pathotypes were isolated less frequently with each of them being present in this study sample at a frequency that was <10%. Pathotype X exhibited an incompatible interaction with all differentials as well as susceptible control variety (IR24) indicating that this pathotype consists of less virulent strains. This pathotype was present in 11/20 sampled states and constituted 4 % of the total Xoo isolates. It is likely that pathotype X is a grouping of strains from other pathotypes that have, for unknown reasons, lost their virulence. Pathotype XI consisted of six isolates collected from Tripura in North-East India and showed a compatible interaction with all the differentials. Except for pathotype XI, all other pathotypes that are compatible with *xa13* are incompatible with *xa5* and those that are compatible with *xa5* are incompatible with *xa13*. Pathotypes V and X are incompatible with both *xa5* and *xa13*. Strains from all pathotypes, except for pathotype XI, are found in the Eastern Indian state of Odisha (Table 2). The pathotype III is the most frequent pathotype in 16/20 states covered in this study. Interestingly, pathotypes that are compatible with *xa13* were not isolated in half of the states that have been surveyed. These include the states of Andhra Pradesh, Karnataka, Maharashtra, Gujarat, Himachal Pradesh, Uttarakhand, Jharkand, Kerala, Manipur and Madhya Pradesh. However, the sample size was limited in some of these states. Although a large number of isolates were collected from the states of Haryana and Punjab, only a few isolates (1 out of 72 isolates and 3 out of 130 isolates respectively) were compatible with *xa13*. In comparison, many of the isolates that are compatible with *xa13* were found in the states of Eastern India, particularly in Odisha and Chhattisgarh (54 out of 152 and 25 out of 193, respectively). 

**Table 2:** Distribution of *X*. *oryzae* pv. *oryzae* pathotypes in different states of India.

State^a^	Pathotypes											**Total isolates**
	**I**	**II**	**III**	**IV**	**V**	**VI**	**VII**	**VIII**	**IX**	**X**	**XI**	
**Andhra Pradesh**	14 (16.5)	4 (4.7)	56 (65.9)	3 (3.5)	5 (5.9)					3 (3.5)		85
**Assam**			1 (14.3)		4 (57.1)			1 (14.3)	1 (14.3)			7
**Bihar**	10 (18.2)	9 (16.4)	12 (21.8)	1 (1.8)	2 (3.6)				21 (38.2)			55
**Chhattisgarh**	32 (16.6)	1 (0.5)	96 (49.7)	4 (2.1)	23 (11.9)		17 (8.8)		8 (4.1)	12 (6.2)		193
**Gujarat**			12 (70.6)		5 (29.4)							17
**Himachal Pradesh**			4 (57.1)	1 (14.3)	2 (28.6)							7
**Haryana**	12 (16.7)	3 (4.2)	32 (44.4)	13 (18.1)	8 (11.1)			1 (1.4)		3 (4.2)		72
**Jharkhand**			6 (100)									6
**Karnataka**	1 (6.7)		10 (66.7)							4 (26.7)		15
**Kerala**	1 (12.5)	2 (25.0)	4 (50.0)	1 (12.5)								8
**Manipur**			2 (66.7)		1 (33.3)							3
**Maharashtra**	17 (38.6)	5 (11.4)	18 (40.9)	3 (6.8)	1 (2.3)							44
**Madhya Pradesh**		1 (14.3)	5 (71.4)	1 (14.3)								7
**Odisha**	26 (17.1)	20 (13.2)	34 (22.4)	10 (6.6)	4 (2.6)	5 (3.3)	22 (14.5)	10 (6.6)	17 (11.2)	4 (2.6)		152
**Punjab**	36 (27.7)	9 (6.9)	59 (45.4)	3 (2.3)	16 (12.3)		1 (0.8)		2 (1.5)	4 (3.1)		130
**Tamil Nadu**	2 (25.0)		4 (50.0)						2 (25.0)			8
**Tripura**	1 (7.1)							3 (21.4)	4 (28.6)		6 (42.9)	14
**Uttar Pradesh**	24 (25.0)	3 (3.1)	36 (37.5)	4 (4.2)	4 (4.2)		14 (14.6)	1 (1.0)	10 (10.4)			96
**Uttarakhand**	27 (39.7)	5 (7.4)	16 (23.5)	8 (11.8)	1 (1.5)					11 (16.2)		68
**West Bengal**	7 (18.9)	1 (2.7)	10 (27.0)	3 (8.1)				2 (5.4)	14 (37.8)			37
												1024

^a^ The number of isolates of each pathotype is provided along with the total number of isolates collected from the state . The numbers in parenthesis indicate % frequency.

**Figure 3:**
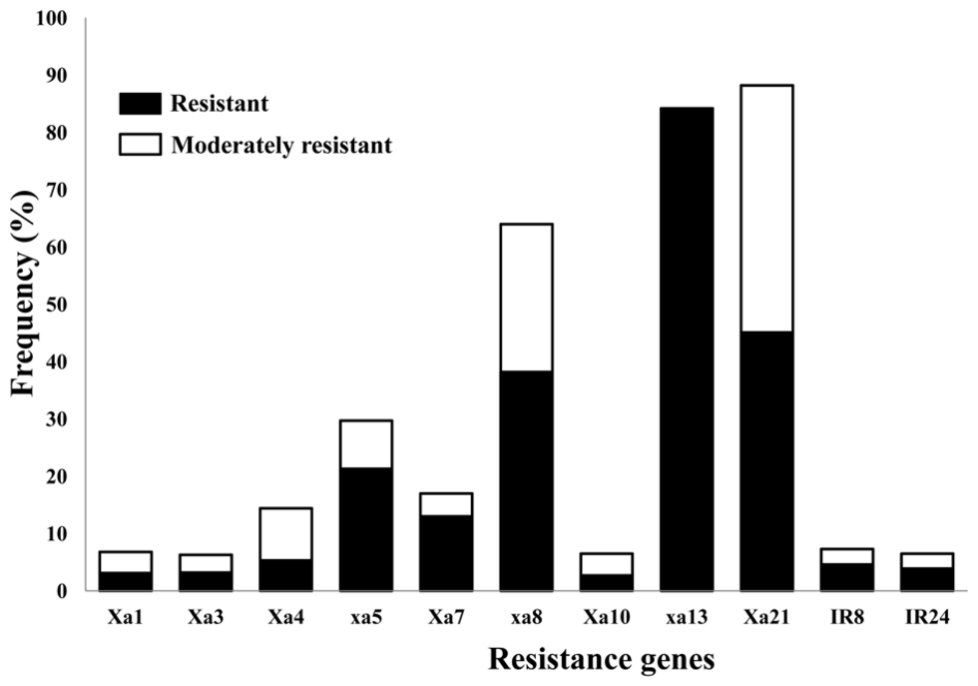
Effectiveness of Xa genes against Indian *X. oryzae* pv. *oryzae* isolates. The x-axis indicates resistance gene and the y-axis indicates the frequency of isolates against which this gene confers resistance. Inoculation of rice leaves with isolates was done as described in Methods and lesion lengths were measured 14 days after inoculation. Lesion lengths <5 cm are considered as resistant, 5 to 10 cm are considered as moderately resistant and >10 cm are considered as susceptible.

Considering the individual R-genes, *Xa21* appeared as the most broadly effective, conferring resistance against 88 % of the isolates, followed in decreasing order by *xa13* (84 %), *xa8* (64 %), *xa5* (30 %), *Xa7* (17 %) and *Xa4* (14 %) (Figure 3). The R-genes *Xa1*, *Xa3* and *Xa10* were not effective against any of the pathotypes, except for those strains belonging to pathotype X. 

The reaction of the isolates against a set of gene pyramid lines carrying several combinations of ‘Xa’ genes was assessed (Table 3). The IRBB52 line which harbours both *Xa4* and *Xa21* genes exhibited a moderate level of resistance against pathotype II and a resistant reaction against pathotype VI. The NILs that carry either one of these individual resistance genes exhibited a susceptible reaction with pathotype II and a moderate level of resistance against pathotype VI. In particular, the reaction with pathotype II indicates that the presence of both of the ‘defeated’ genes leads to an enhanced level of resistance, a phenomenon that has been previously described and termed as quantitative complementation (QC) [3,4,23]. Similarly QC was also observed in IRBB 52, IRBB54 (*xa5* + *Xa21*), IRBB55 (*xa13* + *Xa21*) and IRBB 58 (*Xa4* + *xa13* + *Xa21*) lines which exhibited a moderate level of resistance against pathotype XI as compared to the susceptible reaction in lines carrying the respective single resistance genes. In another example of QC, IRBB54 exhibited a moderate level of resistance towards pathotype IV as compared to the susceptible interaction exhibited by lines carrying either *xa5* or *Xa21*. In an important observation, the IRBB59 line (*xa5* + *xa13* + *Xa21*) is found to exhibit resistance to pathotype XI even though the lines carrying each of the individual genes are susceptible. Interestingly, the IRBB55 line exhibits a susceptible response towards pathotype VI while the line carrying *Xa21* alone exhibits a moderate level of resistance against this pathotype. This suggests that, in the presence of *xa13*, there is a reduction in the level of resistance that is provided by *Xa21* against pathotype VI.

**Table 3:** Reactions of *X. oryzae* pv. *oryzae* pathotypes on *Xa* gene pyramid lines.

**Gene Pyramids** ^b^	**Xoo pathotypes** ^a^										
	**I**	**II**	**III**	**IV**	**V**	**VI**	**VII**	**VIII**	**IX**	**X**	**XI**
**IRBB52 (Xa4 + Xa21**)	R	M^c^	R	S	R	R^c^	R	R	R	R	M^c^
**IRBB54 (*xa5* + *Xa21***)	R	S	R	M^c^	R	R	R	R	R	R	M^c^
**IRBB55 (*xa13* + *Xa21***)	R	R	R	R	R	S^d^	M	R	M	R	M^c^
**IRBB58 (*Xa4* + *xa13* + *Xa21***)	R	R	R	R	R	M	R	R	M	R	M^c^
**IRBB59** (***xa5* + *xa13* + *Xa21***)	R	R	R	R	R	R	R	R	R	R	R^c^
**IRBB60 (*Xa4* + *xa5* + *xa13* + *Xa21***)	R	R	R	R	R	R	R	R	R	R	R^c^

^a^ Based on mean lesion length, the nature of responses were classified into susceptible (S) (above 10 cm), moderately resistant (M) (between 5-10 cm) and resistant (R) (up to 5 cm).

^b^ The Xa genes that are present in the gene pyramid line is indicated in parentheses.

^c^ The gene pyramid lines exhibit more resistance than the lines having the single ‘R’ genes.

^d^ The gene pyramid line exhibits less resistance than the lines having the single ‘R’ genes.

In order to determine the shortest path by which the eleven pathotypes could be derived from each other, we have performed a minimum spanning network analysis (Figure S1) using the pathotyping data from Table 1. We have added, to the different edges (connecting lines) in this network, a hypothetical directionality by assuming that in most cases the evolution of a pathotype would have involved acquisition of the ability to break down a host resistance gene. Pathotype V is incompatible with the *xa5* and the *xa13* resistance genes. Therefore, pathotype V could be an ancestral pathotype from which strains that are compatible with *xa5* or *xa13* could have evolved by acquisition of the ability to breakdown these resistance genes. The hypothesized change from pathotype V to pathotype III might have involved acquisition of the ability to break down the *xa5* resistance gene as well as *Xa4*. Pathotype II might have arisen from pathotype I through acquisition of the ability to break down the *Xa21* resistance gene or from pathotype IV through acquisition of the ability to break down the *xa8* resistance gene. Pathotype XI may have arisen from pathotype II through acquisition of the ability to breakdown the *xa13* resistance gene. The hypothesized change from pathotype V to pathotype VII might have involved acquisition of the ability to break down the *xa13* resistance gene. The hypothesized change from pathotype VII to VI would have resulted in susceptibility to the *Xa11* resistance gene and the change from pathotype VII to VIII would have required acquisition of the ability to break down the *xa8* resistance gene. In comparison to pathotype VII, pathotype VIII is also avirulent on the *Xa7* resistance gene. Pathotype IX may have arisen from pathotype VIII through acquisition of the ability to breakdown the *Xa4* resistance gene.

### RFLP analysis of *Xanthomonas oryzae* pv. *oryzae* strains

**Figure 4:**
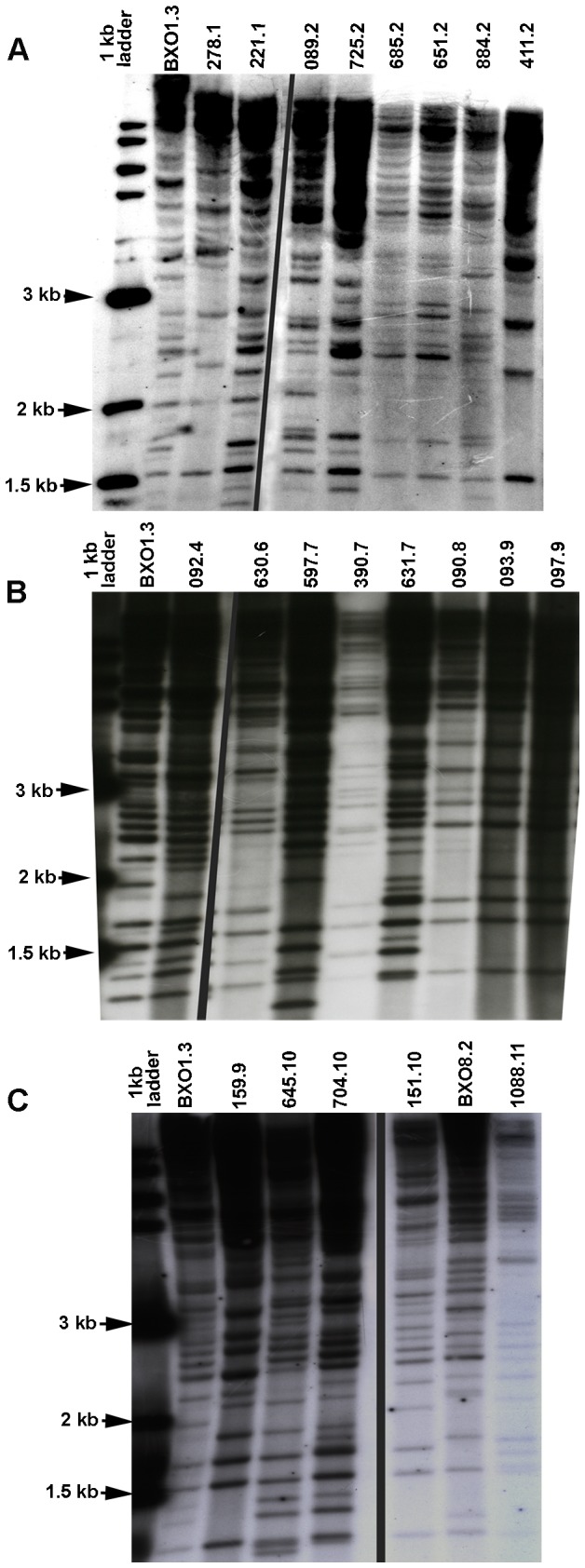
Restriction Fragment Length Polymorphism analysis of *X. oryzae* pv. *oryzae* strains. Genomic DNA was isolated from 52 *X. oryzae* pv. *oryzae* strains and southern analysis was performed using the IS*1112* probe as described in Methods. These 52 strains included 50 strains from eleven pathotypes identified in this study as well as *X. oryzae* pv. *oryzae* strains BXO1 and BXO8 from the study of Yashitola et al (13). Twenty different haplotypes were identified and at least one representative of each haplotype is shown. The IXO strain number is indicated on each lane along with pathotype. A one kb DNA ladder was added as a size marker. *X. oryzae* pv. *oryzae* strain BXO1 is loaded in the second lane of each blot for comparison. Each strain was analyzed at least three times, and 29 bands that were consistently visible in all the three replicates were used for scoring.

RFLP based genotyping of 50 isolates belonging to 11 different pathotypes was done by scoring 29 bands obtained by using IS*1112*, an Xoo insertion sequence element, as the DNA fingerprinting probe. Five strains each of pathotypes V, VIII, IX and X; four strains each of pathotypes I, III, IV and VI; six strains each of pathotypes II and VII and two strains of pathotype XI were analyzed (Table 4). Two other strains, namely BXO1 and BXO8, which were pathotyped and genotyped in an earlier study [13] were also included as reference strains. The RFLP and phylogenetic analysis using UPGMA revealed that 20 haplotypes were present amongst the 50+2 isolates that were studied (Figures 4 and Figure S2). The most frequent RFLP haplotype consisted of 13 isolates including the following: 4 strains belonging to pathotype III, each of which was isolated from four different states (Andhra Pradesh, Chhattisgarh, Punjab and West Bengal), 4 strains belonging to pathotype V isolated from different states (Andhra Pradesh, Bihar, Maharashtra and Punjab) as well as two strains each of pathotypes I and IV and one strain from pathotype X. This indicates that pathotypes III as well as V consisted of genetically closely related strains which had dispersed to several widely distributed locations in India. The BXO1 strain, which was included as a reference strain, is found to have the same RFLP haplotype as these 13 strains. The study of Yashitola et al. (1997) had indicated that strains with the same RFLP haplotype as BXO1 were widely distributed in India. The BXO1 strain belongs to pathotype III. Isolates of pathotype VI were isolated from a specific region in India (the state of Odisha) and all the four genotyped isolates of this pathotype have a single haplotype. Isolates of pathotype IX that were isolated from Odisha (East), Uttar Pradesh (North) and Tamil Nadu (South) clustered together in the dendrogram. In contrast isolates belonging to pathotypes I, II and X were found to be composed of genetically diverse strains as they grouped into different clusters. Genotyping of two strains belonging to pathotype XI indicated that they belong to a haplotype which is quite divergent from the rest of the strains. Also, the BXO8 strain which had been previously found to be a very diverse strain [13], is also found to be an outlier in this study. 

**Table 4:** List of representative *X. oryzae* pv. *oryzae* strains that were used for Restriction Fragment Length Polymorphism Analysis.

**Sl. No.**	**Isolate**	**Location**	**Rice cultivar^a^**	**Year of isolation**	**Pathotype**
1	IXO221	Andhra Pradesh	MTU1010	2005	I
2	IXO278	Uttar Pradesh	Arize 6444	2005	I
3	IXO812	Andhra Pradesh	Arize 6516	2007	I
4	IXO675	Punjab	Pusa 44	2007	I
5	IXO89	Uttarakhand	NA	2004	II
6	IXO884	Maharashtra	MTU 7029	2008	II
7	IXO685	Kerala	NA	2006	II
8	IXO725	Maharashtra	H93025	2007	II
9	IXO411	Chhattisgarh	Mahamaya	2006	II
10	IXO651	Punjab	Pusa Basmati 1	2006	II
11	IXO639	Punjab	Pusa 44	2006	III
12	IXO281	Andhra Pradesh	MTU1001	2005	III
13	IXO74	Chhattisgarh	Arize 6444	2004	III
14	IXO189	West Bengal	H93024	2005	III
15	IXO92	Punjab	PR118	2005	IV
16	IXO367	Uttarakhand	NA	2006	IV
17	IXO35	Chhattisgarh	IRBB 5	2004	IV
18	IXO98	Himanchal Pradesh	NA	2005	IV
19	IXO99	Andhra Pradesh	BPT 5204	2005	V
20	IXO518	Gujarat	Arize 6444	2006	V
21	IXO414	Bihar	Arize 6129	2006	V
22	IXO134	Maharashtra	NA	2005	V
23	IXO220	Punjab	Arize 6111	2005	V
24	IXO620	Odisha	MTU 7029	2005	VI
25	IXO644	Odisha	NA	2005	VI
26	IXO621	Odisha	Surendra	2005	VI
27	IXO630	Odisha	NA	2005	VI
28	IXO222	Odisha	NA	2005	VII
29	IXO599	Uttar Pradesh	Arize 6444	2005	VII
30	IXO597	Uttar Pradesh	Arize 6444	2005	VII
31	IXO631	Odisha	NA	2005	VII
32	IXO390	Odisha	NA	2005	VII
33	IXO792	Chhattisgarh	MTU 7029	2007	VII
34	IXO90	Uttar Pradesh	BPT 5204	2004	VIII
35	IXO493	Odisha	NA	2006	VIII
36	IXO169	Uttar Pradesh	Arize 6444	2005	VIII
37	IXO141	Odisha	Surendra	2005	VIII
38	IXO365	Odisha	MTU 7029	2005	VIII
39	IXO93	Uttar Pradesh	Indrasan	2005	IX
40	IXO608	Odisha	MTU 7029	2007	IX
41	IXO603	Odisha	MTU 7029	2007	IX
42	IXO159	Uttar Pradesh	Roopali	2005	IX
43	IXO97	Tamil Nadu	NA	2005	IX
44	IXO53	Karnataka	NA	2004	X
45	IXO151	Haryana	HKR 47	2005	X
46	IXO842	Chhattisgarh	Arize 6444	2007	X
47	IXO645	Odisha	Arize Tej	2005	X
48	IXO704	Punjab	Rasi 7001	2007	X
49	IXO1088	Tripura	MTU 7029	2009	XI
50	IXO1104	Tripura	MTU 7029	2009	XI

^a^ Rice cultivar from which the strain was isolated; NA indicates that the information is not available

**Figure 5:**
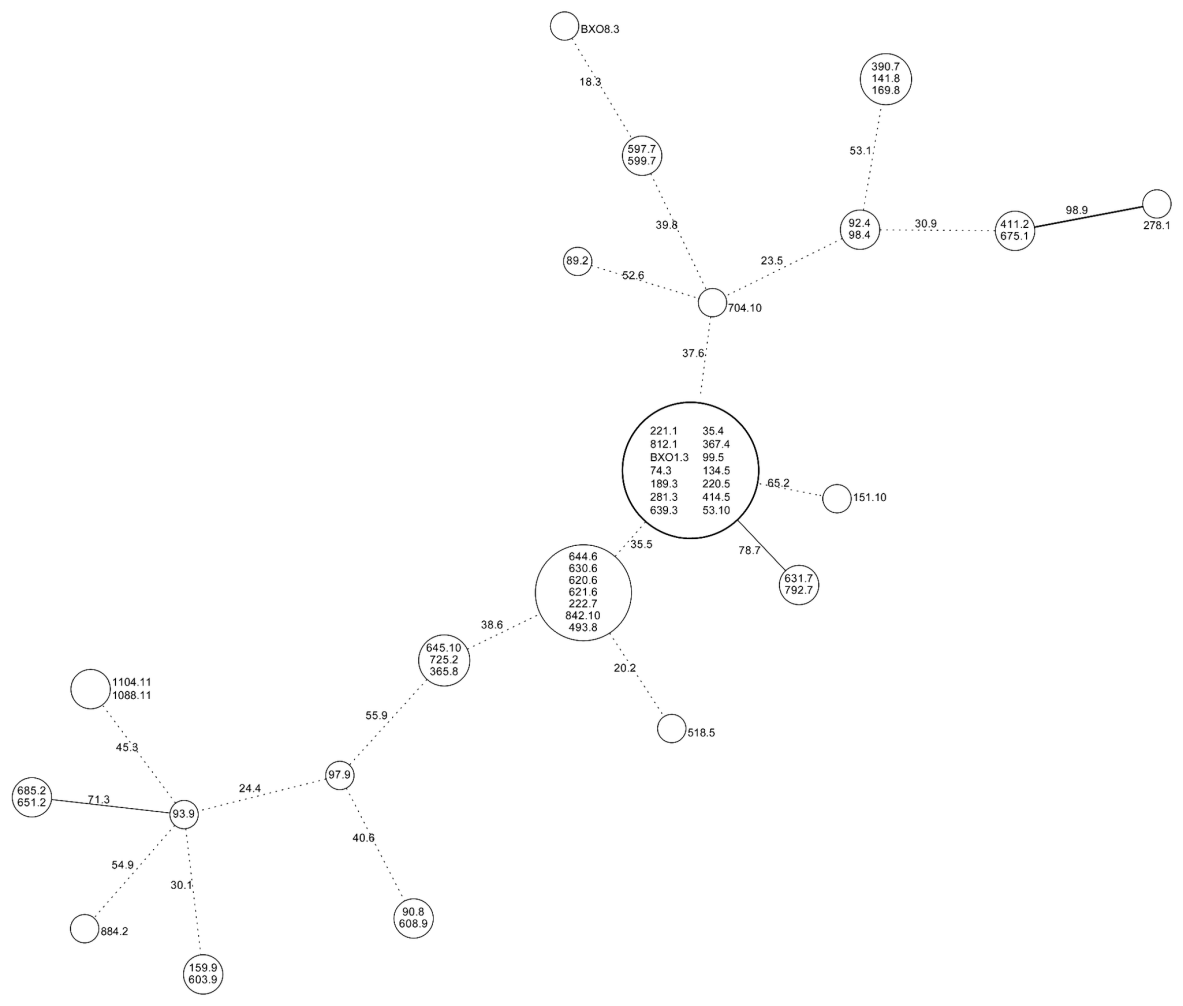
Phylogenetic relationships of *X. oryzae* pv. *oryzae* strains analyzed using a Minimum spanning tree. A minimum spanning tree was drawn using the RFLP data. The digits in the edges represent percentage boot strap values obtained after 1000 iterations. A solid bold edge indicates a bootstrap value over 90%; solid line indicates a value over 70% and a dotted line indicates a value below 70%.

Additional phylogenetic analysis was performed, using the same RFLP fragment data, by constructing a minimum spanning tree which can help in hypothesizing on ancestral or emergent RFLP haplotypes (Figure 5). The RFLP haplotype representing 13 strains (14 including BXO1) occupies an approximately central position in the tree. As indicated above, this is a widely distributed RFLP haplotype in India and includes strains from five different pathotypes. These observations, taken together, suggest that this might be an ancestral RFLP haplotype. Interestingly, this RFLP haplotype includes 4 strains of pathotype V which is hypothesized to be an ancestral pathotype. An RFLP haplotype that is shared by 7 strains (four of pathotype VI and one each of pathotypes VII, VIII and X) is connected to this haplotype and could be derived from it. Also, the postulated ancestral RFLP haplotype is connected by an edge having a strong bootstrap value to an RFLP haplotype shared by two pathotype VII strains suggesting that the latter might be an emergent haplotype. A different RFLP haplotype (strain IXO93.9) is connected to five different haplotypes. Support for this grouping is provided by the observation that two nodes representing three strains belonging to pathotype IX are connected to IXO93.9. Interestingly, two pathotype II strains that share an RFLP haplotype are connected to IXO93.9 through an edge that has a high bootstrap value. As was observed in the tree made by UPGMA, the pathotype II strains appear at multiple places in the minimum spanning tree indicating that they are a diverse set of strains. Further studies on pathotype II strains using additional rice differentials might reveal significant pathological differences between them. 

## Discussion

Based on the study sample of 1024 strains, and the differential rice lines used in this study, eleven different pathotypes of this bacterium have been identified. Although a number of pathotypes were identified, pathotype XI isolated from the Eastern Indian state of Tripura was very unique in that it was compatible with all the tested *Xa* genes. Fortunately, the gene pyramid line carrying *Xa21*, *xa13* and *xa5* is resistant to this strain. In view of the possible dispersal of this pathotype to other locations in India, efforts should be made to deploy these three resistance genes in the genetic background of important Indian rice cultivars. 

Genotype analysis, using the IS*1112* probe, carried out on a subset of 50 strains that are drawn from the 11 pathotypes (plus the reference strains BXO1 and BXO8) revealed 20 RFLP haplotypes indicating that the genetic base of the Indian Xoo population is fairly diverse. At the same time, the bootstrap values for many of the clusters were not significant. Multiple RFLP probes, PCR based finger printing methods and genotyping using single nucleotide polymorphisms (SNPs) might be needed to delineate the phylogenetic relationships between these strains in a more accurate manner. Using the same RFLP data, a phylogenetic tree was also constructed using minimum spanning tree analysis. One particular RFLP haplotype shared by thirteen different isolates plus BXO1 could be an ancestral haplotype as it is centrally located in the tree and five different pathotypes have this haplotype. 

Although it might be considered as being too simplistic, we have tried to use the data in Table 1 to generate a minimum path by which these eleven pathotypes could have arisen. As pathotype V strains are incompatible with both of the *xa5* and *xa13* disease resistance genes, pathotypes that are compatible with either one or the other of these two resistance genes could have arisen by the acquisition of specific TAL (transcription activator-like) effectors by an ancestral pathotype V strain. The *Xa5* gene encodes transcription factor TFIIAγ and is presumably needed by Xoo TAL effectors to upregulate specific host susceptibility genes [24–26]. The *xa5* disease resistance gene probably does not support this role of TAL effectors in up-regulating expression of host susceptibility factors. Xoo strains that can breakdown *xa5* mediated resistance are postulated to produce TAL effector/s that can utilize an alternate TFIIAγ gene in rice [25]. Therefore a pathotype (such as pathotype III) that can breakdown *xa5* mediated resistance could have arisen from an ancestral pathotype V like strain by acquisition of TAL effector/s that can utilize the alternate TFIIAγ. Pathotypes I, II, III and IV differ from pathotype V in their ability to overcome *xa5* as well as *Xa4* mediated resistance. We speculate that there might have been some bridging strains that were able to overcome *xa5* mediated resistance but were unable to overcome *Xa4* mediated resistance. The previously described pathotype Ia which is reported to be incompatible with *Xa4* could be such a pathotype. 

The *xa13* disease resistance gene represents a mutation in the promoter of *Os8N3*, a host susceptibility gene that encodes a sugar transporter (SWEET protein) [27–29]. Xoo strains that overcome *xa13* mediated resistance are capable of producing a TAL effector that can upregulate the expression of an alternate SWEET gene of the host [30]. It is possible that pathotype VII could have arisen from pathotype V by acquisition of such a TAL effector. Although it is speculative, we postulate that the first step in the evolution of Pathotypes VI, VII, VIII and IX (that can all breakdown *xa13* mediated resistance) would have been the acquisition of such a TAL effector by an ancestral pathotype V like strain. 

Does the minimum spanning tree in Figure 5 provide any support for the notion that many of the current day Indian pathotypes of Xoo could have arisen from an ancestral pathotype V like strain? The RFLP haplotype that has been suggested above to be an ancestral haplotype includes four out of five pathotype V strains analyzed in this study. Moreover, several pathotype III, pathotype IV and pathotype I strains share the same RFLP haplotype. Therefore, it is possible that pathotypes III, IV and I could have evolved from an ancestral pathotype V strain. Figure 5 also suggests that RFLP haplotypes that represent pathotype VII could have emerged from this supposedly ancestral RFLP haplotype. Therefore, it is possible that pathotype VII strains could have arisen from an ancestral pathotype V like strain. Figure 5 also suggests that at least some pathotype VIII and IX strains could have arisen from pathotype VII strains. 

The suggestion that pathotype V might be an ancestral pathotype from which other pathotypes arose through acquisition of compatibility against either *xa5* or *xa13* provides an explanation for the observation that the pathotypes that are compatible with *xa5* are incompatible with *xa13* and vice versa. The exceptions include pathotype X strains which are incompatible with all the rice lines tested in this study. We suggest that pathotype X strains might have lost virulence as growth ceased in dried lesions of rice leaves. Spontaneous loss of virulence has been previously reported from aging cultures of Xoo [31]. Pathotype XI strains are compatible with both *xa5* and *xa13* disease resistance genes; one possible way in which this could have occurred is through acquisition of the ability to overcome *xa13* mediated resistance by a pathotype (such as pathotype II) that is already compatible with *xa5*. An alternative possibility that pathotype XI strains arose through acquisition of the ability to overcome *xa5* mediated resistance by a strain that is already compatible with *xa13* cannot be ruled out and indeed does have some support from the minimum spanning tree analysis (Figure 5). 

A number of strains belonging to pathotypes III and V had the same haplotype even though they were isolated from widely separated locations. This suggests the possibility that they have been dispersed through seed. Pathotype IX strains cluster together in the dendrogram indicating that they are closely related strains. These strains were isolated from three geographically separated states in India; an observation that is again suggestive of the possibility of dispersal through seed. The BXO1 strain of Xoo has been previously described by Yashitola et al. (1996) as belonging to pathotype Ib which belongs to a widely distributed lineage of this pathogen in India. In that study, the same RFLP haplotype as that of the BXO1 strain was found in 15/67 strains that had been analyzed with the IS*1112* probe and these strains were widely distributed in India. The results presented here indicate that the BXO1 strain has the same RFLP haplotype as the most prevalent RFLP haplotype found in this study. The current study confirms the results of Yashitola et al. (1996) and indicates that strains with the same RFLP haplotype as BXO1 continue to be widely distributed in India. The same RFLP haplotype had also been found to be present in a majority of Xoo isolates collected from a wild rice species (*Oryza nivara*) growing naturally in Southern India and it had been suggested that this haplotype might have transferred from wild rice to cultivated rice [32].

Among the Xoo resistance genes studied, *Xa21* was found to be most effective towards Indian Xoo strains followed by *xa13* and *xa8*. All three genes appear to be good candidates to be deployed in Indian rice cultivars. The current study also throws light on the suitability of different *Xa* gene combinations for deployment in India. Since the most effective ‘R’ genes are *Xa21* and *xa13*, a combination of these two genes will be a natural choice. However, as per this study, a number of Indian Xoo strains that are compatible with *xa13* are incompatible with *xa5*. Therefore, a gene combination of *Xa21*, *xa13* and *xa5* might be an added advantage. This gene combination is also effective against pathotype XI. Two different research groups in India have pyramided the *Xa21* and *xa13* resistance genes into the popular rice varieties Pusa Basmati-1 and Samba Mahsuri [5,6]. In the latter work, the *xa5* resistance gene was also incorporated along with *Xa21* and *xa13* to create a three gene pyramid line. 

The present study also shows examples of a phenomenon called quantitative complementation (QC) wherein the presence of two different ‘Xa’ genes provides an increased level of resistance as compared to either of the single resistance genes. In this study, as described in Table 3, several such examples of QC could be observed. Interestingly, we have also observed an example of antagonistic interaction between *Xa* genes, wherein the pyramid of *Xa21* and *xa13* was less resistant than the line having *Xa21* alone. This antagonistic interaction was observed when this gene pyramid was inoculated with isolates belonging to pathotype VI. A similar example of pathotype specific antagonistic interaction between *Xa21* and *xa13* has been previously described [33]. A negative interaction between *Xa21* and *xa5* only in the genetic background of a specific rice cultivar has also been previously described [34]. These examples highlight the need to take into consideration the possible effects of genetic background of the cultivar and pathotype prevalence in the region while making decisions on deployment of *Xa* gene pyramid lines in bacterial blight affected rice growing regions. 

In summary, 1024 Xoo strains were collected from 20 different states in India and subjected to pathotyping and RFLP analysis. This has provided interesting insights into the genetic and pathotypic diversity of Indian strains of Xoo. However, several states were either not sampled or were sampled rather sparsely. Also, very little information is available about the TAL effectors and the host susceptibility genes that are used by Indian strains of Xoo to break down rice resistance genes such as *xa5*, *xa13*, etc. Future studies should be aimed at addressing these issues. 

## Supporting Information

Figure S1 **A Minimum spanning network of eleven different *X. oryzae* pv. *oryzae* pathotypes.** The network was developed using data from Table 1 as indicated in methods. The direction of the hypothetical change is indicated by the arrow. The resistance gene against which compatibility is gained (+) or lost (-) during the change from one pathotype to another is indicated. Dotted lines are given when alternate edges are possible. The digits given on the dotted edges indicate the percentage of minimum spanning trees having that particular edge.
(TIF)

Figure S2 **Dendrogram of 52 Indian strains of *X. oryzae* pv. *oryzae* derived from restriction fragment length polymorphism analysis using the IS*1112* repeat element.** The dendrogram was constructed and bootstrap values calculated as described in methods. The digits in the nodes represent percent boot strap values after 2000 iterations. The IXO number along with the pathotype is indicated for each strain. The scale bar represents genetic divergence. The BXO1 and BXO8 strains previously described by Yashitola et al. 1997 (13) were included for comparison.
(TIF)
