# Supplementary figures and images for: Pathotype and Genetic Diversity amongst Indian Isolates of Xanthomonas oryzae pv. oryzae
Source: PLoS One. 2013 Nov 29;8(11):e81996. doi: 10.1371/journal.pone.0081996 (PMC3843720; doi:10.1371/journal.pone.0081996)

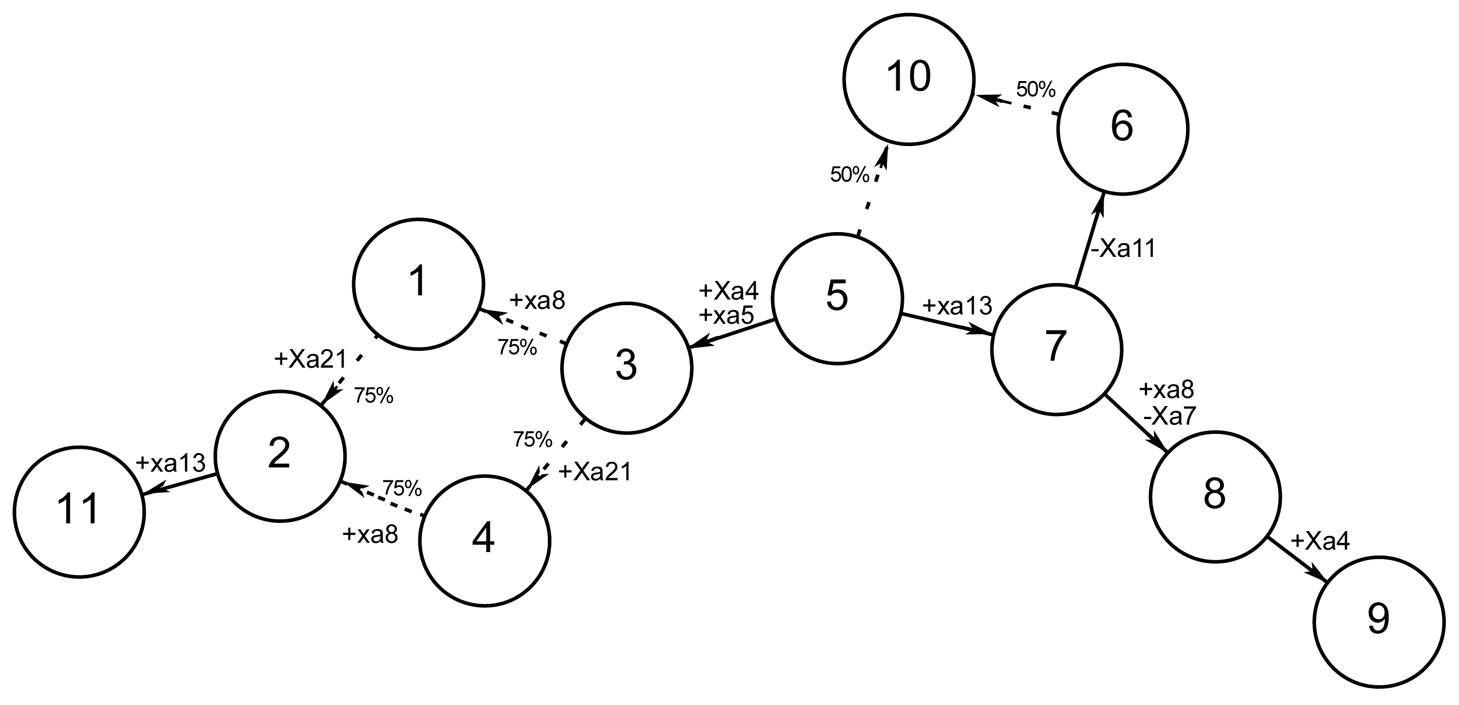

Supplement: Figure S1 — A Minimum spanning network of eleven different X. oryzae pv. oryzae pathotypes. The network was developed using data from Table 1 as indicated in methods. The direction of the hypothetical change is indicated by the arrow. The resistance gene against which compatibility is gained (+) or lost (-) during the change from one pathotype to another is indicated. Dotted lines are given when alternate edges are possible. The digits given on the dotted edges indicate the percentage of minimum spanning trees having that particular edge. (TIF) [file pone.0081996.s001.tif]

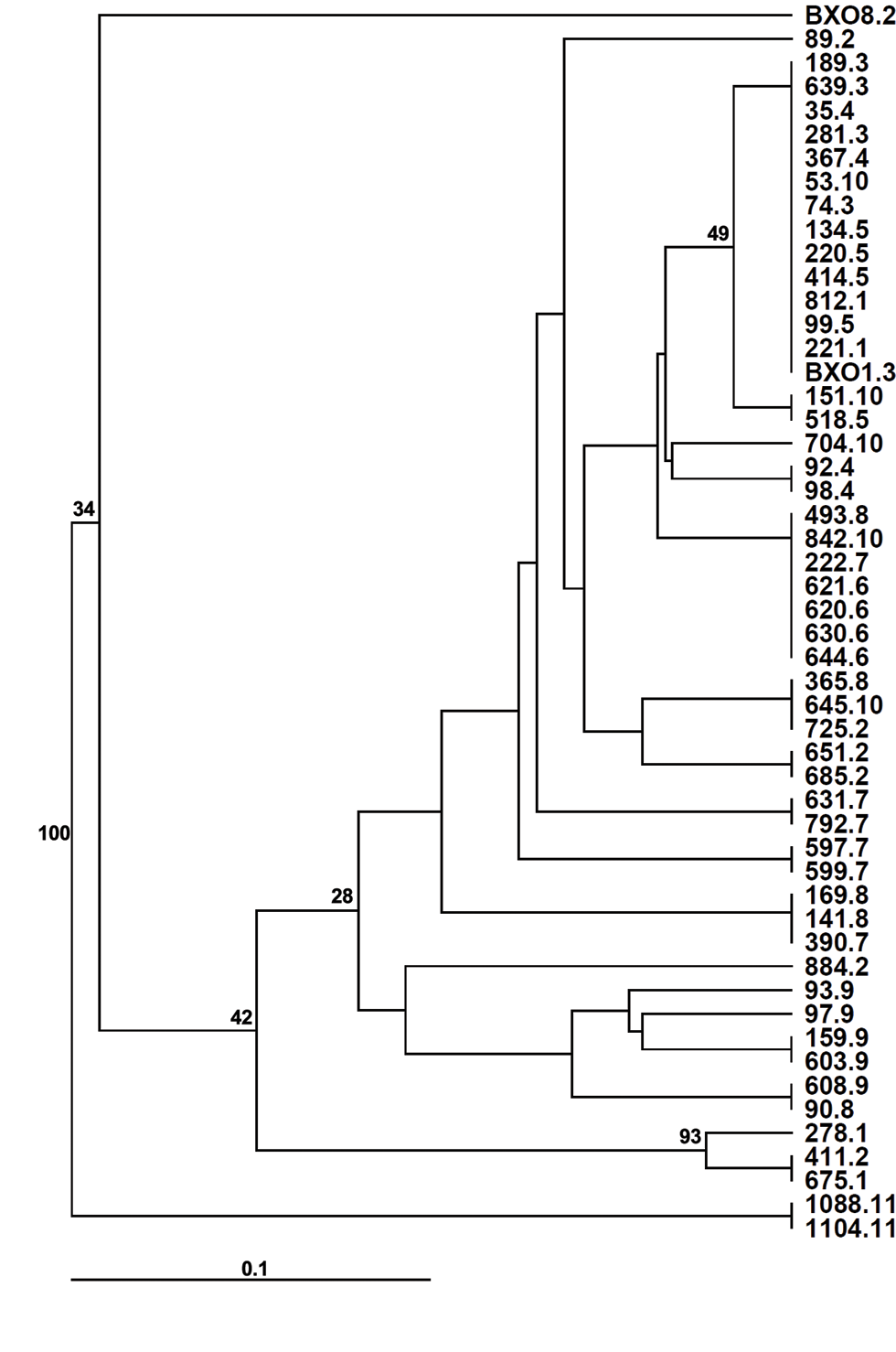

Supplement: Figure S2 — Dendrogram of 52 Indian strains of X. oryzae pv. oryzae derived from restriction fragment length polymorphism analysis using the IS1112 repeat element. The dendrogram was constructed and bootstrap values calculated as described in methods. The digits in the nodes represent percent boot strap values after 2000 iterations. The IXO number along with the pathotype is indicated for each strain. The scale bar represents genetic divergence. The BXO1 and BXO8 strains previously described by Yashitola et al. 1997 (13) were included for comparison. (TIF) [file pone.0081996.s002.tif]
